# Sex differences in classic congenital adrenal hyperplasia: a multicenter, real-world analysis

**DOI:** 10.3389/fendo.2026.1788502

**Published:** 2026-04-27

**Authors:** Chiara Simeoli, Davide Ferrari, Marianna Minnetti, Nicola Di Paola, Ylenia Alessi, Maria Elena Aloini, Andrea M. Isidori, Rosario Pivonello, Renata Simona Auriemma, Annamaria Colao

**Affiliations:** 1 Dipartimento di Medicina Clinica e Chirurgia, Sezione di Endocrinologia, Diabetologia, Andrologia e Nutrizione, Università “Federico II” di Napoli, Naples, Italy; 2 Department of Experimental Medicine, Sapienza University of Rome, Rome, Italy; 3 Department of Biomedical, Dental and Morphological and Functional Imaging Sciences, University of Messina, Messina, Italy; 4 Endocrinology, Department of Clinical and Molecular Medicine, Sant’Andrea University Hospital, Sapienza University of Rome, Rome, Italy; 5 Centre for Rare Diseases (Endo-ERN Accredited), Policlinico Umberto I, Rome, Italy; 6 Unesco Chair for Health Education and Sustainable Development, “Federico II” University, Naples, Italy

**Keywords:** adiposity, androstenedione, congenital adrenal hyperplasia (CAH), gender, glucocorticoids, obesity, sex

## Abstract

**Background:**

The phenotypic variability of classic congenital adrenal hyperplasia (CAH) demands tailored management to optimize disease control and attenuate long-term consequences. Although sex-specific differences have been recognized, real-world evidence in adults remains limited and inconsistent. Therefore, this study aimed to compare the clinical, metabolic, and hormonal profiles of adult male and female patients with classic CAH and identify sex-specific predictors of biochemical control.

**Methods:**

In this multicenter, real-world analysis, records from adult CAH patients followed at four Italian tertiary centers, focusing on anthropometry, glucocorticoid (GC) therapy, biochemical and hormonal parameters, were retrospectively collected. Whole cohort and sex-stratified predictors of uncontrolled disease were then identified.

**Results:**

Overall, 123 patients [77 females, 30.5 (24–37) years; 46 males, 32 (23–40) years] were enrolled. Males showed greater overweight/obesity rates (75.6% vs. 46.7%, p = 0.001), BMI (26.6 vs. 24.4 kg/m^2^, p = 0.003) and visceral adiposity indices [waist-to-height ratio (WHtR) 0.55 vs. 0.51, p = 0.006]. GC formulations and total daily doses were comparable between the sexes. Uncontrolled disease occurred in 26.2% of males and 17.6% of females (p = 0.271). WHtR was the strongest predictor of androstenedione (β = 0.311, p = 0.003). Sex-stratified analysis revealed that visceral adiposity and glucose metabolism abnormalities were critical predictors of poor control among males but not females.

**Conclusions:**

Adult males with CAH may face a vicious cycle of adiposity-driven metabolic and hormonal dysregulation, hindering disease control; however, estrogens may counteract these effects in well-controlled females. These results advocate for prioritizing metabolic health for long-term CAH management.

## 1 Introduction

Congenital adrenal hyperplasia (CAH) is a group of rare autosomal recessive disorders characterized by defective adrenal steroidogenesis, most commonly (95%) due to pathogenic variants of the *CYP21A2* gene encoding 21-hydroxylase (P450c21) (1, 2). Classic CAH presents with cortisol deficiency, whereas its salt-wasting (SW) forms present concomitant aldosterone deficiency, which can lead to life-threatening adrenal crises in newborns. Impaired cortisol biosynthesis lifts the negative feedback on the hypothalamus and pituitary gland, leading to excess adrenocorticotropic hormone (ACTH) production, adrenal hyperplasia, and androgen overproduction. Glucocorticoid (GC) therapy, introduced in the 1950s, transformed survival by replacing cortisol and suppressing excess androgen production (3). However, GC therapy often requires supraphysiological doses, which predispose patients to long-term adverse effects (4).

Balancing hormonal control while minimizing GC-related consequences is critical for reducing the complex clinical and hormonal consequences affecting individuals with CAH according to age, developmental stage, and sex, although maintaining such balance remains challenging. Indeed, classic CAH is the most frequent etiology of primary adrenal insufficiency in early childhood (5, 6) and is the leading cause of sexual development disorders in 46, XX individuals (7). Both sexes may experience precocious puberty and accelerated growth (8, 9), which reduce their final height (10). At puberty, females often present with hirsutism, acne, and menstrual irregularities, resembling polycystic ovary syndrome (PCOS) (11–13), whereas up to 90% of males develop testicular adrenal rest tumors (TARTs), compromising fertility and gonadal function (14–18). In adulthood, overexposure to GCs increases cardiometabolic, skeletal, and cognitive risk, ultimately impairing quality of life (QoL) (4, 19, 20). Sex differences may influence the natural course and complications of CAH. Indeed, previous studies have reported sex differences in comorbidities, QoL, and neuropsychological outcomes (21–25), warranting deeper, large-scale investigations that could support more personalized and effective long-term management strategies (23).

Hence, the current study aimed to evaluate sex-related differences in classic CAH presentation, biochemical profile, treatment, and long-term complications by analyzing a substantial body of real-world clinical and biochemical data from multiple Italian referral centers.

## 2 Materials and methods

### 2.1 Study design and ethics approval

A retrospective, multicenter study was performed by collecting real-world data from the following four university hospitals across Italy that specialized in the management of CAH, with specific expertise in transition care and adult endocrinology: “Federico II” University of Naples, “Sapienza” University of Rome; “Sant’Andrea” University Hospital of Rome; and “G. Martino” University Hospital of Messina. These centers actively collaborated within the multicenter observational registry GONADIS, approved by local ethics committees (Protocol number 252/19). This study was conducted following Good Clinical Practices and the principles of the Declaration of Helsinki. All study subjects provided written informed consent for the use of their clinical data.

This study included adult patients with a genetically confirmed diagnosis of classic CAH due to 21-hydroxylase [both SW and simple-virilizing (SV) forms] who were receiving stable GC therapy for at least 3 months, in the absence of stress dosing for at least 2 weeks and were receiving regular follow-up at the participating centers (26). Patients with non-classic CAH or other enzyme defects and those not receiving GCs were excluded.

### 2.2 Measurements

Data on sex, age, and clinical history, focusing on CAH subtype, disease duration, and adrenal crises, were retrospectively extracted from the medical records. Therapeutic GC regimens were reviewed, with GC therapy classified as modified-release hydrocortisone (MR-HC), immediate-release hydrocortisone (IR-HC), dual-release hydrocortisone (DR-HC), cortisone acetate (CA), prednisone (PRED), or dexamethasone (DEX). All dosages were standardized through conversion to hydrocortisone equivalents (HCeq) and adjusted to body surface area (BSA). Additionally, the use of mineralocorticoid therapy with fludrocortisone was recorded.

Body composition parameters, including height, weight, and body mass index (BMI) categorized according to the World Health Organization criteria (27), waist circumference, and blood pressure were retrospectively collected. To assess visceral adiposity, the following indexes were calculated: waist-to-height ratio (WHtR); conicity index (C-Index); and lipid accumulation product (LAP) as previously described (28–31). The following formulas were used:


WHtR=waist(cm)/height(cm)

$$\text{C}-\text{Index}=\text{waist}(\text{m})/[0.109\times \sqrt{\frac{\text{weight}\;(\text{kg})}{\text{height}\;(\text{m})}}]$$

LAP(males)=[waist(cm)–65]×triglycerides(mmol/L)

LAP(females)=[waist(cm)–58]×triglycerides(mmol/L)

A WHtR cut-off ≥ 0.5 is widely used to define increased cardiometabolic risk, while for the C-Index, values >1.20 in males and >1.18 in females indicate a higher risk of metabolic abnormalities (32). No specific cut-off has been established for LAP, as it was originally proposed as a continuous marker of lipid overaccumulation rather than a categorical variable, with higher values indicating greater lipid accumulation (31). 

All biochemical parameters, including serum levels of electrolytes, blood count, glucose metabolism, including fasting plasma glucose, fasting insulin, glycated hemoglobin (HbA1c) and homeostatic model assessment- insulin resistance (HOMA-IR), and lipid profile, as well as hormonal parameters, including 17-hydroxyprogesterone (17OHP), androstenedione (A4), dehydroepiandrosterone sulfate (DHEAS), testosterone (T), ACTH, and renin were measured using validated methods aligned to international standards. Hormonal results were normalized as ratios to the upper limit of normal (ULN) to reduce inter-assay variability. Details on the analytical techniques employed for hormone measurements are specified in the Supplementary File 1. The prevalence of comorbidities associated with CAH and GC therapy, including overweight, obesity, arterial hypertension, diabetes mellitus or other glucose abnormalities, dyslipidemia, infertility, hypogonadism, psychiatric disorders, and bone fragility was also assessed. Specifically, insulin resistance was estimated *via* HOMA-IR with cut-off value ≥ 2.5 indicating insulin resistance (33). Infertility was defined in both sexes as the inability to achieve pregnancy after at least 12 months of regular, unprotected sexual intercourse, consistent with the World Health Organization (WHO) and the American Society for Reproductive Medicine (ASRM) guidelines (34, 35). Hypogonadism was clinically defined as T levels <12 nmol/L or T replacement therapy in males, following the clinical practice guidelines of the Italian Society of Andrology and Sexual Medicine (SIAMS) and the Italian Society of Endocrinology (SIE), as well as the European Academy of Andrology (EAA) guidelines (36, 37). In the attempt to distinguish T of adrenal or gonadal origin, A4/T ratio was derived in male patients. In line with previous reports, an A4/T <0.5 supports a gonadal origin of T, while a ratio >1 indicates an adrenal origin and usually poorer disease control (38–40). In premenopausal female patients not on oral contraceptives, hypogonadism was defined by prolonged amenorrhea (>6 months) with low follicular-phase estradiol levels according to the clinical evaluation of the treating endocrinologist. This approach was adopted because, in patients with CAH, circulating estradiol may partly derive from peripheral aromatization of adrenal androgens and therefore may not always accurately reflect primary ovarian function (41).

Disease control was defined as A4 levels below the ULN and was combined with total daily GC dose to classify patients into four categories: controlled disease with physiological GC replacement (A4 ≤ ULN, GC dose ≤ 15 mg/m^2^/day); controlled disease with supraphysiological GCs (A4 ≤ ULN, GC dose > 15 mg/m^2^/day); uncontrolled disease with physiological GCs (A4 > ULN, GC dose ≤ 15 mg/m^2^/day); uncontrolled disease with supraphysiological GCs (A4 > ULN, GC dose >15 mg/m^2^/day).

### 2.3 Statistical analysis

Continuous variables were reported as mean ± standard deviation or median with interquartile range (IQR, 25–75), based on the Shapiro–Wilk test to assess data distribution. Categorical variables were expressed as frequencies and percentages. Comparisons between the sexes were conducted using independent t-tests or Mann–Whitney U tests for continuous variables and the chi-squared or Fisher’s exact tests for categorical variables. Anthropometric comparisons were adjusted for age and GC dose using analysis of covariance (ANCOVA) models. Similarly, GC dose comparisons were adjusted for BSA. In all ANCOVA models, Bonferroni correction was applied.

A linear regression model was created to explore associations between A4 and clinical, biochemical, and hormonal variables, while adjusting for age, sex, GC dose (in HCeq), and diagnosis type (SW/SV). Multicollinearity was assessed *via* variance inflation factors (VIF). Associations between A4 control and each variable were analyzed using univariate logistic regression. For each outcome and each variable separately, regression analysis was conducted in both the full cohort and after stratifying the patients according to sex, with results reported as odds ratios (OR) and 95% confidence intervals (CI). Subsequent multivariate logistic regression analyses were performed, including clinically essential covariates such as age, sex, type of CAH, and daily GC dose. Non-normally distributed variables were log-transformed before inclusion in regression analyses. All data were analyzed using SPSS version 28 (SPSS, Inc., Cary, NC, United States) and GraphPad Prism 10. A p value of <0.05 indicated statistical significance, whereas 0.05 < p < 0.1 indicated possible trends.

## 3 Results

### 3.1 General characteristics

The study cohort consisted of 123 adult patients with CAH [46 males (37.4%) and 77 females (62.6%)], with a median age of 31 (24–38) years and a median disease duration of 29 (21–37) years, which were comparable between the sexes (**Table 1**). While SW-CAH was the more prevalent CAH subtype in both sexes, SV-CAH was more frequently detected in females compared to males (17.4% vs 36.4%, p = 0.025 for CAH etiology distribution). Moreover, 19.6% of males and 27.3% of females reported a history of adrenal crises (p = 0.229), with no significant difference in the number of episodes across both sexes [2 (1–4) vs. 3 (2–5), p = 0.244] (**Table 1**).

**Table 1:** General disease characteristics of the cohort.

		Male patients (n= 46)		Female patients (n= 77)	P-value*
General characteristics					
Age, (years)		32 (23-40)	30.5 (24-37)		0.450
Disease duration, (years)		31.0 (21.5-39.0)	28.0 (21-35.3)		0.255
History of adrenal crises, (prevalence)		19.6%	27.3%		0.229
Salt-wasting CAH, (prevalence)		82.6%	63.6%		0.025
Simple virilizing CAH, (prevalence)		17.4%	36.4%		
Glucocorticoid and mineralocorticoid therapy^a^					
Modified-release Hydrocortisone	- Prevalence	60.84%	54.5%		0.493
	- Daily dose, mg	25 (20-30)	20 (20-30)		0.736
Immediate-release Hydrocortisone	- Prevalence	36.96%	29.9%		0.417
	- Daily dose, mg	30 (25-35)	17.5 (15.0-20.0)		0.089
Dual-release Hydrocortisone	- Prevalence	2.2%	9.1%		0.132
	- Daily dose, mg	25	20 (10-30)		0.730
Cortisone Acetate	- Prevalence	0.0%	5.2%		0.116
	- Daily dose, mg	–	37.5 (28.1-46.9)		–
Prednisone	- Prevalence	0.0%	1.3%		0.438
	- Daily dose, mg	-	7.5		-
Dexamethasone (add-on therapy)	- Prevalence	6.5%	6.5%		0.995
	- Daily dose, mg	0.38 (0.07-0.38)	0.25 (0.19-0.62)		0.545
Total HCeq daily GC dose, mg		27.5 (30-35)	20 (15-30)		0.018^b^ / 0.656^b^
BSA-adjusted HCeq daily GC dose, mg/m^2^		14.3 (11.3-18.1)	13.6 (10.2-17.9)		0.533
Stress dose use in the last 3 months, prevalence		4.3%	2.6%		0.777
Fludrocortisone acetate, prevalence		71.7%	63.6%		0.235
Total fludrocortisone daily dose, mg		0.11 (0.09-0.20)	0.10 (0.06-0.15)		0.070^b^ / 0.912^b^
BSA-adjusted fludrocortisone daily dose, mg/m^2^		0.06 (0.05-0.09)	0.06 (0.04-0.09)		0.920

Continuous data are shown as median (IQR 25-75). Categorical data are shown as percentages. Sex comparisons are performed by Mann–Whitney U or Chi-squared test. ^a^For each GC formulation, the comparison mean daily dose is corrected for BSA in an ANCOVA model. ^b^The first p-value refers to the unadjusted comparison, while the second p-value refers to the BSA-adjusted comparison using an ANCOVA model. CAH, congenital adrenal hyperplasia; HCeq, hydrocortisone-equivalent; GC, glucocorticoid; BSA, body surface area.

#### 3.1.1 GC and mineralocorticoid therapy

GC and mineralocorticoid therapies are detailed in **Table 1**. Collectively, over a half of the cohort (56.9%) received MR-HC, with no differences in prevalence (p = 0.493) or total daily dose (p = 0.736) between males and females. IR-HC was used by 32.5% of the included patients at similar rates across both sexes (p = 0.417), although males tended to receive higher doses (p = 0.089). Other formulations were used less frequently: DR-HC in 6.5% of patients (one male, seven females), CA (5.2%) and PRED (1.3%) only in females, and evening DEX in 6.5% of both (three males and five females, in an add-on therapy) (p = 0.995). The median daily BSA-adjusted HCeq GC dose was 13.7 (10.5–18.1) mg/m^2^ and did not differ according to sex [p = 0.533; ANCOVA mean estimated difference, 1.0 mg (95% CI: −3.3 - 5.2), p = 0.656] (**Table 1**). Stress dosing in the prior 3 months was low (3.3%) and comparable (p = 0.777) across both sexes. Fludrocortisone was used by 66.7%, again with similar rates (p = 0.235) and doses (BSA-adjusted: p = 0.920) between sexes (**Table 1**).

### 3.2 Analysis of body composition, comorbidities and biochemical parameters

#### 3.2.1 Body composition parameters

Males had a higher BMI than females [26.6 vs. 24.4 kg/m^2^, p = 0.003], a difference that remained significant after adjusting for age and GC dose (mean estimated difference +2.67 kg/m^2^, 95% CI: 0.80–4.52, p = 0.005) (**Table 2**). Visceral adiposity indexes were consistently higher in males, including WHtR (0.55 vs. 0.51, p = 0.006), C-Index (1.269 vs. 1.179, p <0.001) and LAP (22.6 vs. 14.7, p = 0.014) (**Table 2**). After adjusting for age and GC dose, all differences remained significant (p = 0.007 for WHtR, p = 0.008 for C-index and p = 0.048 for LAP). Similarly, males showed a higher prevalence of pathological WHtR (73.7% vs 55.2%) and C-index (65.0% vs 49.2%) scores.

**Table 2:** Sex differences in body composition and comorbidities.

	Male patients (n= 46)	Female patients (n= 77)	P-value
Anthropometric parameters			
Height (cm)	167 (164 – 171)	158 (152 – 162)	<0.001
BMI (Kg/m^2^)	26.6 (24.8-31.3)	24.4 (22.3-27.9)	0.003
Waist Circumference (cm)	79.0 (73.0-90.0)	94.5 (84.8-101.3)	<0.001
Waist-to-height ratio	0.55 (0.50-0.62)	0.51 (0.46-0.55)	0.006
LAP	22.6 (12.8-38.5)	14.7 (9.2-25.3)	0.014
C-Index	1.269 (1.155-1.322)	1.179 (1.106-1.235)	<0.001
Comorbidities			
Obesity (BMI ≥ 30 Kg/m^2^)	33.4%	20.0%	0.079
Overweight (25 Kg/m^2^ ≤ BMI < 30 Kg/m^2^)	42.2%	26.7%	0.060
Overweight or Obesity (BMI ≥25 Kg/m^2^)	75.6%	46.7%	0.001
Arterial Hypertension	15.2%	5.2%	0.124
Diabetes mellitus	2.2%	3.9%	0.602
Insulin-resistance	21.7%	23.4%	0.509
Impaired fasting glucose	4.3%	7.8%	0.366
Dyslipidemia	23.9%	16.9%	0.341
Infertility	23.1%	4.8%	0.006
Hypogonadism	30.4%	5.8%	<0.001
Successful ART	6.5%	0.0%	0.022
Psychiatric complications	0.0%	3.9%	0.187
Osteopenia	22.5%	21.9%	0.943
Osteoporosis	7.0%	5.5%	0.744
Fragility fractures	2.4%	0%	0.199

Continuous data are shown as median (IQR 25-75). Categorical data are shown as percentages. Sex comparisons are performed by Mann–Whitney U or Chi-squared test. BMI, body mass index; LAP, Lipid Accumulation Product; C-Index, Conicity Index; ART, Assisted Reproductive Technology.

#### 3.2.2 Comorbidities

Collectively, the prevalence of overweight or obesity was higher in males (75.6% vs. 46.7%, p = 0.001). Specifically, overweight was detected in 42.2% of males and 26.7% of females, whereas obesity was detected in 33.4% of males and 20.0% of females. Arterial hypertension was slightly more frequent in males, although not significantly. No differences in diabetes mellitus, other glucose abnormalities, or dyslipidemia were observed between males and females (**Table 2**). Infertility was more common in males (23.1% vs. 4.8%, p = 0.006), with three cases of successful assisted reproductive technology. Hypogonadism was more frequent in males (p < 0.001), with one patient (7.1%) requiring T therapy. Male patients showed a median A4/T of 0.34 (0.14-1.15) with 51.3% showing a ratio <0.5, suggesting gonadal origin of T. Conversely, 28.2% of patients showed an A4/T ratio >1, indicating higher adrenal contribution to T levels and poorer disease control. The prevalence of other comorbidities was similar between sexes (**Table 2**).

#### 3.2.3 Biochemical parameters

**Figure 1:**
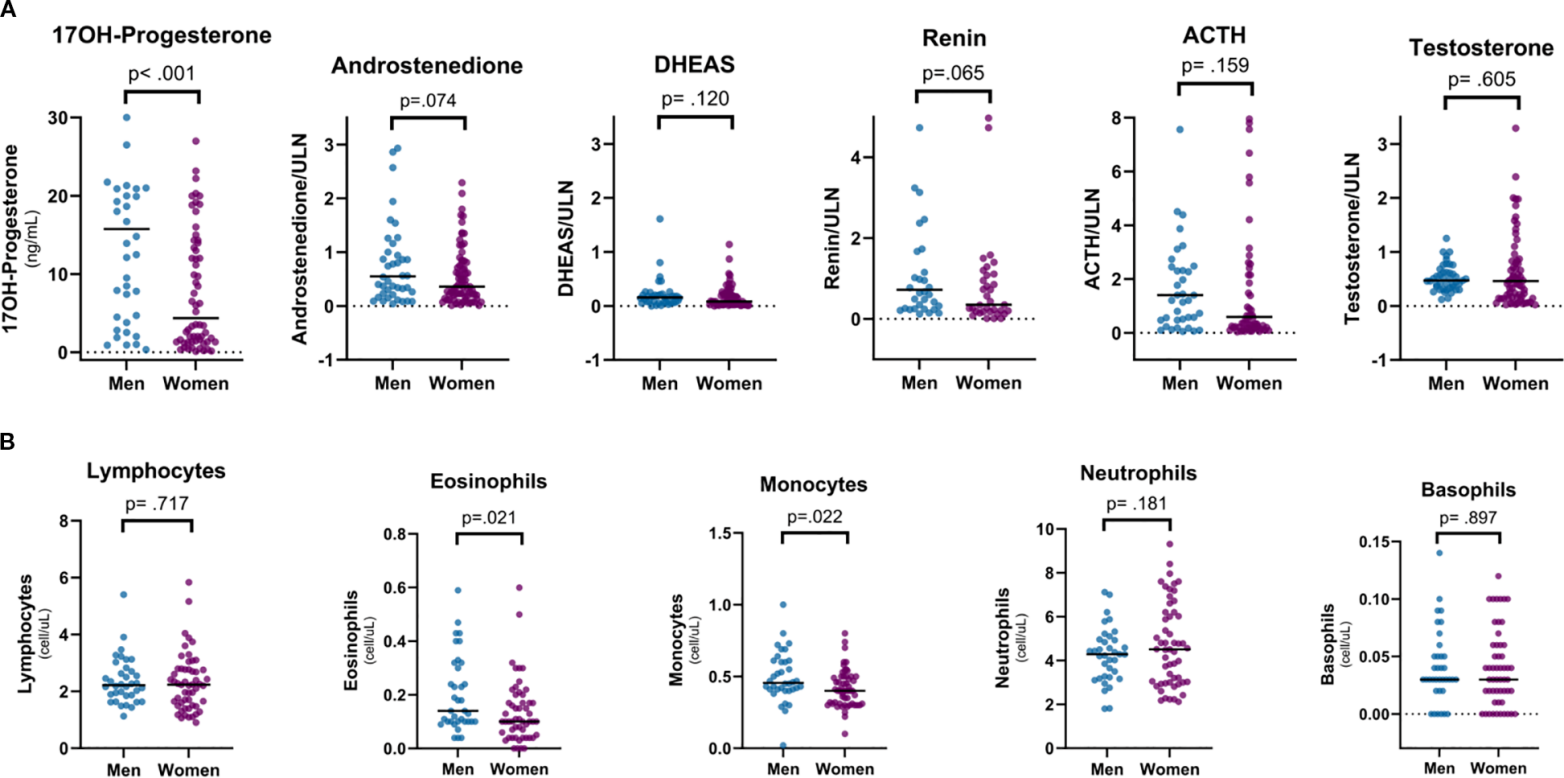
Sex differences in hormone profiles **(A)** and circulating leucocytes **(B)**. Data are presented as scattered dot plots with line at median. Blue dots represent male subjects; purple dots represent female subjects.

Electrolyte levels were normal and comparable between the sexes. Males showed higher red blood cell (p <0.001 for both), eosinophil (p = 0.021), and monocyte (p = 0.022) counts, with no differences in basophil and neutrophil counts (**Table 3** and **Figure 1**).

**Table 3:** Sex differences in biochemical parameters.

	Male patients (n= 46)	Female patients (n= 77)	P-value
Serum Electrolytes			
Sodium, (mEq/L)	139.7 ± 2.5	138.9 ± 2.6	0.096
Potassium, (mEq/L)	4.3 ± 0.4	4.3 ± 0.5	0.876
Calcium, (mg/dL)	9.5 ± 0.4	9.5 ± 0.5	0.533
Phosphorus, (mg/dL)	3.6 ± 0.6	3.6 ± 0.7	0.758
Blood count			
Red Blood Cells (10^6^/µL)	5.2 ± 0.4	4.6 ± 0.4	<0.001
Hemoglobin (g/dL)	15.3 ± 1.2	13.7 ± 1.1	<0.001
White Blood Cells (10^3^/µL)	7.5 ± 2.0	7.7 ± 2.0	0.587
Lymphocytes (10^3^/µL)	2.4 ± 0.8	2.3 ± 1.0	0.717
Neutrophils (10^3^/µL)	4.3 ± 1.2	4.7 ± 1.9	0.181
Eosinophils (10^3^/µL)	0.20 ± 0.14	0.14 ± 0.12	0.021
Basophils (10^3^/µL)	0.04 ± 0.03	0.04 ± 0.03	0.897
Monocytes (10^3^/µL)	0.49 ± 0.18	0.41 ± 0.13	0.022
Platelets (10^3^/µL)	239 ± 56	266 ± 74	0.195
Glucose and lipid metabolism*			
Fasting plasma Glucose, (mg/dL)	85.5 ± 6.6	82.5 ± 9.4	0.107
Fasting Insulin, (µU/mL)	9.9 (6.2-12.3)	9.3 (6.6-13.7)	0.837
HOMA-IR	2.09 (1.17-2.55)	1.81 (1.19-2.96)	0.641
HbA1c, (mmol/mol)	33 (30-37)	32 (31-34)	0.249
Total Cholesterol, (mg/dL)	167 ± 30	179 ± 30	0.039
LDL Cholesterol, (mg/dL)	100 (82-116)	92 (79-112)	0.295
HDL Cholesterol, (mg/dL)	47 (39-63)	63 (51-78)	<0.001
Triglycerides (mg/dL)	87 (67-105)	73 (54-99)	0.047

Data are presented as mean ± SD or median (IQR 25–75), according to distribution. Sex comparisons were performed using Student’s t-test or Mann–Whitney U test, as appropriate. *Excluding patients with diabetes mellitus. HOMA-IR, homeostasic model assesment - insulin resistance; HbA1c, glycated hemoglobin; LDL, low density lipoprotein; HDL, high density lipoprotein.

In patients without diabetes mellitus, fasting glucose (83.6 ± 9.6 mg/dL) and insulin [9.5 (6.4–13.4) µU/mL] were similar in both sexes. Accordingly, HOMA-IR [2.10 (1.19–2.70)] and HbA1c [33 (31–36) mmol/mol] did not significantly differ between both sexes (p = 0.641 and 0.249). Females had higher total and high-density lipoprotein (HDL) cholesterol levels (p = 0.038 and <0.001, respectively), whereas males had higher triglyceride levels (p = 0.047) (**Table 3**).

When performing sex-based comparisons stratified by disease subtype (**Supplementary Table 1**), we found that sex differences were preserved in the SW-CAH subgroup, whereas they were less evident in SV-CAH. Specifically, within the SV-CAH subgroup, the only adiposity parameter showing a significant sex difference was C-index (p = 0.035). Regarding hormonal parameters, 17OHP showed a trend toward higher levels in males, while A4 did not differ between sexes (**Supplementary Table 1**).

### 3.3 Analysis of disease control and its determinants

Males had higher 17OHP levels [15.8 (4.6–21.6) vs. 3.5 (1.3–14.0) ng/mL, p <0.001] and tended to have higher A4 levels [A4/ULN ratio 0.55 (0.23–1.18) vs. 0.36 (0.15–0.80), p = 0.074]. DHEAS levels were similar between both sexes [DHEAS/ULN ratio 0.16 (0.06–0.20) vs. 0.08 (0.03–0.19), p = 0.120], whereas ACTH levels [ACTH/ULN ratio 1.39 (0.49–3.18) vs. 0.59 (0.19–2.95), p = 0.159] and renin levels [renin/ULN ratio 0.72 (0.26–2.39) vs. 0.35 (0.18–1.11), p = 0.065] tended to be higher in males, although not significantly (**Figure 1**).

**Figure 2:**
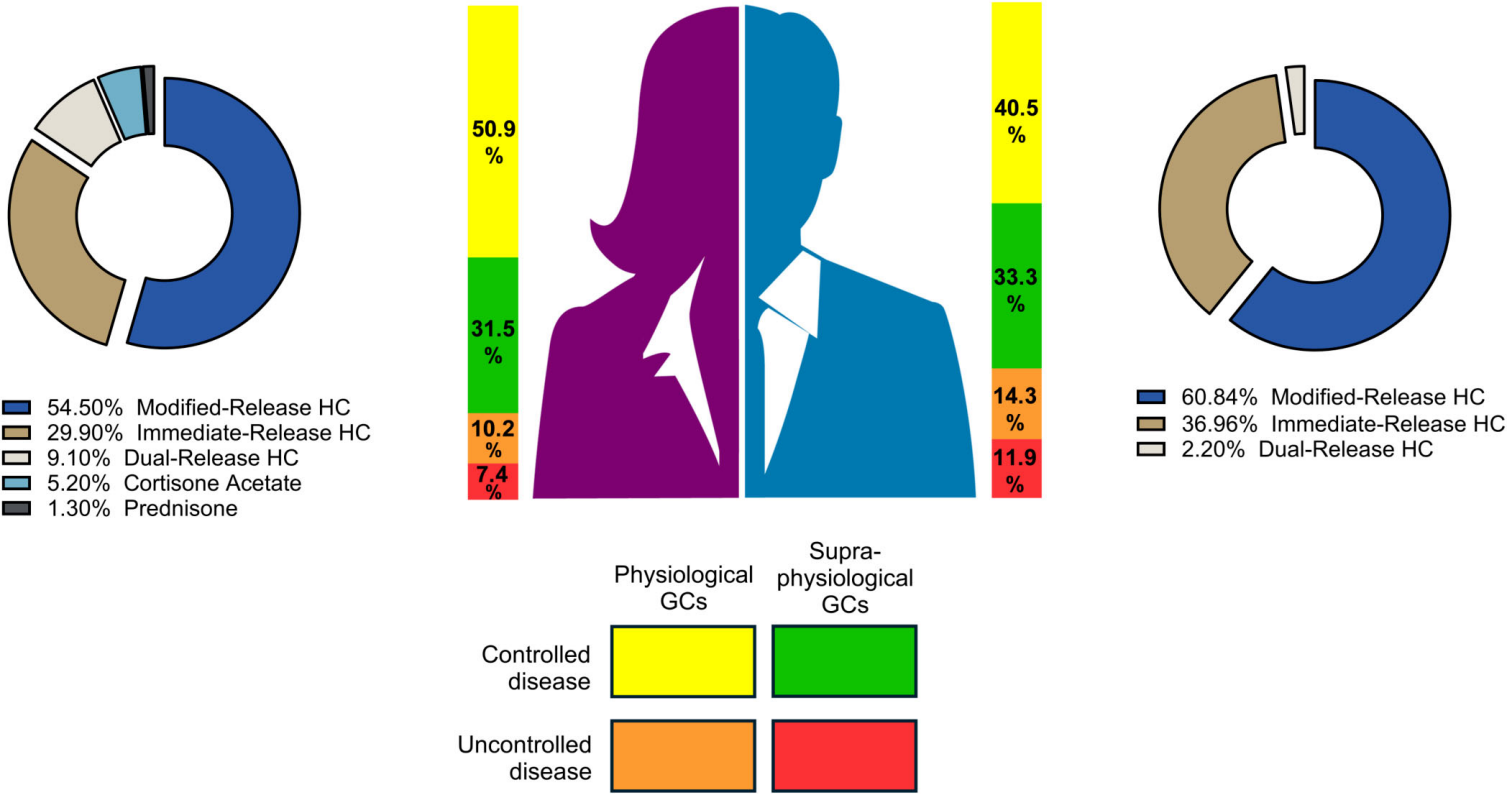
Comparison of disease control status and type of GC replacement between male and female patients. Visual representation of sex differences in glucocorticoid formulation use and disease control.

No significant differences in disease control were observed between the sexes, with 73.8% of males and 82.4% of females having A4 levels below the ULN (p = 0.271). Among the controlled patients, 40.5% of males and 50.9% of females achieved normal A4 levels with physiological GC doses (p = 0.198), whereas 33.3% of males and 31.5% of females required supraphysiological GC doses (p = 0.305). Among the uncontrolled patients, 14.3% of males and 10.2% of females received physiological GC doses (p = 0.377), whereas 11.9% of males and 7.4% of females received supraphysiological GC doses (p = 0.324) (**Figure 2**).

A multiple linear regression was performed to assess the role of WHtR in predicting A4/ULN levels, adjusting for sex, GC dose (in HCeq), CAH type, and age. WHtR was the only significant predictor of A4 levels (B = 5.682; 95% CI for B: 2.863–8.500; p < 0.001), whereas other clinically essential covariates were not. Collinearity diagnostics indicated no relevant multicollinearity among predictors (tolerance 0.856–0.942, VIF 1.061–1.168). Clinically, a 0.01 increase in WHtR corresponded to an approximate 6% ULN increase in A4, holding the other covariates constant. Accordingly, multivariate backward logistic regression analysis revealed that overweight/obesity status [step 1: B = 1.154, p = 0.042, OR = 3.17 (95% CI: 1.04–9.63)], but not diagnosis type and sex, was a significant predictor of uncontrolled A4. Similarly, 17OHP levels did not show any association with total GC dose in HCeq (B= -0.450, p = 0.955).

**Figure 3:**
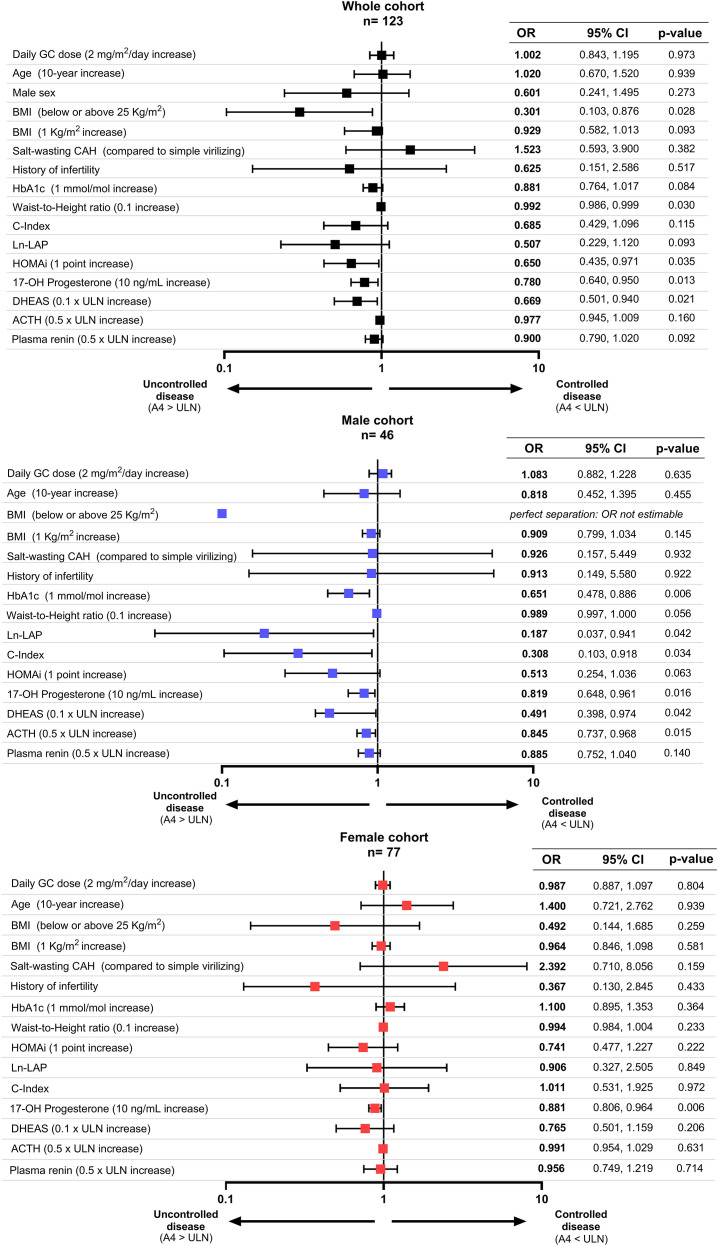
Univariate logistic regression analysis of controlled versus uncontrolled androstenedione levels. Odds ratios (OR) with 95% confidence intervals (CI) are shown for each variable analyzed separately. Black squares indicate the whole cohort, blue squares the male cohort, and red squares the female cohort. Error bars correspond to 95% CI. GC, glucocorticoid; BMI, body mass index; CAH, congenital adrenal hyperplasia; HbA1c, glycated hemoglobin; HOMA-i, homeostatic model assesment- insulin resistance; LAP, lipid accumulation product; C-index, conicity index; DHEAS, dehydroepiandrosterone sulfate; ACTH, adrenocorticotrpic hormone.

The results of the univariate analysis considering the whole cohort and males and females separately are represented in **Figure 3**. All male patients with a BMI ≥ 25 kg/m^2^ had uncontrolled disease, which resulted in complete separation and prevented the estimation of OR and 95% CI for overweight/obesity status. WHtR was also negatively associated with disease control (OR = 0.992 per 0.1 increase, 95% CI: 0.986–0.999, p = 0.030), with the C-index and LAP showing similar but no significant trends. HOMA-IR also inversely predicted A4 control (OR = 0.650, 95% CI: 0.435–0.971, p = 0.035), with HbA1c showing a borderline effect (OR = 0.881, 95% CI: 0.764–1.017, p = 0.084) (**Figure 3**).

After sex stratification, visceral adiposity markers [C-Index (OR = 0.308, 95% CI: 0.103–0.918, p = 0.034) and LAP (OR = 0.187, 95% CI: 0.037–0.941, p = 0.042)] predicted poor control only in males, with WHtR showing a similar trend (OR = 0.989 per 0.1 increase, 95% CI: 0.997–1.000, p = 0.056). Associations between disease control and glucose homeostasis also persisted in males, with significant findings observed for HbA1c (OR = 0.651, 95% CI: 0.478–0.886, p = 0.006) and trends observed for HOMA-IR (OR = 0.513, 95% CI: 0.254–1.036, p = 0.063) but were absent in females (**Figure 3**).

17OHP was the only steroid consistently predicting poorer A4 control in both males (OR = 0.819, 95% CI: 0.648–0.961, p = 0.016) and females (OR = 0.881, 95% CI: 0.806–0.964, p = 0.006). Other hormonal parameters showed sex-specific patterns. DHEAS levels predicted uncontrolled disease (OR = 0.491 per 0.1 × ULN increase, 95% CI: 0.398–0.974, p = 0.042) in males but not in females (OR = 0.765 per 0.1 × ULN increase, 95% CI: 0.501–1.159, p = 0.206). Similarly, ACTH levels were linked to poor control only in males (OR = 0.845 per 0.5 × ULN increase, 95% CI: 0.737–0.968, p = 0.015). Plasma renin levels showed no significant association with disease control in either group (**Figure 3**).

In the multivariate logistic regression model including HOMA-IR, WHtR, sex, age, and CAH subtype as covariates, WHtR (OR = 0.93, 95% CI: 0.85–1.01, p = 0.095) and HOMA-IR (OR = 0.71, 95% CI: 0.43–1.17, p = 0.176) were not found to be independent predictors of disease control, but consistent negative associations with disease control were retained in the model with comparable effect sizes. Collinearity diagnostics excluded significant overlap. In the logistic regression model including an interaction term, the product HOMA-IR × WHtR was identified as a significant predictor of uncontrolled A4 levels (B = –0.010, p = 0.006, OR = 0.99, 95% CI: 0.982–0.997), whereas sex and CAH subtype were no longer identified as significant predictors.

## 4 Discussion

This multicenter study suggests that visceral adiposity and insulin resistance are associated with disease control in classic CAH, particularly in males. Findings of the current study indicate that adiposity was not only a consequence of long-term GC exposure but also an active contributor to hormonal dysregulation. This association appears to be sexually dimorphic, with the modulatory effects of estrogens on adipose tissue distribution likely being protective among premenopausal females.

Recently, the management of classic CAH has significantly evolved, especially regarding treatment strategies. Looking ahead, non-steroidal medications are expected to ease androgen control and reduce GC overexposure (42–44). However, several aspects of CAH physiopathology remain unclear, with patients showing high variability in disease severity, adrenal crises, long-term effects on metabolism and cardiovascular health, bone fragility, and impaired QoL.

Precision medicine approaches (45) have proven efficacious in identifying apparent determinants of several diseases. Accordingly, the current study aimed to explore whether a better understanding of sex differences could help decode the complex framework of CAH.

Males displayed higher rates of overweight and obesity than did females, reaching a combined rate of 75.6% compared to the 46.7% in females. Moreover, BMI, WHtR, and other markers of visceral obesity were higher in males. This sex difference was more pronounced than that in the general Italian population (46, 47).

Previous studies have reported variable obesity prevalence in CAH (10.3–70%), with conflicting results regarding sex differences (21). Although several studies reported worse BMI and body composition in males than in females (24, 48, 49), others describe the inverse pattern (23, 50). One such study including 87 patients showed a higher obesity prevalence in females than in males (46.8% vs. 27.5%) (51). Notably, in that cohort, females also exhibited poorer disease control than did males (elevated A4 and 17OHP), indicating a potential interplay between metabolic status and disease control in CAH.

In support of this link, data of the current study showed that adiposity parameters were associated with elevated A4, with WHtR showing the most consistent association. WHtR has been identified as a sex-independent marker of metabolic risk and can therefore serve as a valuable tool for assessing obesity (52, 53). In multivariable models, a significant interaction between WHtR and HOMA-IR suggests that central adiposity and insulin resistance jointly relate to biochemical control of CAH. These results highlight the potential role of visceral adiposity as a determinant of disease control in CAH. Unlike subcutaneous fat, visceral fat is metabolically active and pro-inflammatory, promoting insulin resistance and blunting GC receptor sensitivity, thereby exacerbating hypothalamic–pituitary–adrenal (HPA) axis overactivity (54, 55). In CAH, visceral adiposity-induced HPA axis overactivity may explain the worse disease control observed in overweight/obese patients and why higher GC doses may not always promote better hormonal control in the current or previous studies (21, 56). Nonetheless, these associations were restricted to males, consistent with the protective role of estrogens against fat distribution and metabolism (57–59). However, severe androgen excess or inadequate treatment may override this protection, shifting fat distribution toward visceral patterns similar to that observed in males and explaining the conflicting results obtained in previous studies (60). Indeed, sex dimorphism in CAH is intrinsically ambiguous given that hormonal profiles may overlap. Females often show androgen excess and hypoestrogenism, whereas males may develop hypogonadism due to multiple mechanisms, including primary gonadal failure due to TARTs and hypothalamic-pituitary-gonadal axis suppression due to chronic excess of adrenal androgens or GC overtreatment.

The sexually dimorphic effects of GCs on metabolism and adiposity adds an additional layer of complexity. For instance, sexual hormones appear to influence the adipose tissue expression of 11β-hydroxysteroid dehydrogenase type 1 (11β-HSD1), which regenerates active cortisol from inactive cortisone. In particular, androgens upregulate 11β-HSD1 in female rats (61), whereas estrogens downregulated it in male and ovariectomized female rodents (62, 63). In humans, 11β-HSD1 is similarly overexpressed among postmenopausal females and those with PCOS, supporting the protective metabolic role of estrogens and the detrimental effects of androgens against GCs (64–66).

When stratifying the cohort into SW-CAH and SV-CAH, in the former, differences in metabolic parameters and hormonal control were consistent with those observed in the overall cohort. In contrast, in SV-CAH, these differences appeared attenuated. This may be explained by the substantial reduction in sample size, particularly in males (eight). Interestingly, in this subgroup, a smaller sex-related difference in visceral adiposity was paralleled by a similar trend in hormonal control, further supporting a potential link between these two aspects in CAH. However, the interplay between adiposity, metabolic dysregulation, and HPA axis activity, as well as the specific role of genotype, warrants further investigation given that metabolic control may be crucial in the management of this complex disease (21, 67).

The current study confirmed the role of 17OHP as a consistent marker of disease control in both sexes. Our results showed that a threshold of 12.5 ng/mL corresponded to normal A4 in 95% of the patients, aligning with previous findings (56). Conversely, DHEAS was generally below one-fifth of the ULN in both sexes and only predicted A4 control in males. Although 21-hydroxylase deficiency should theoretically elevate DHEAS, its levels often remain within range or are suppressed by GCs (68), reflecting a shift in 17,20-lyase activity, which favors the conversion of 17OHP to A4 and 11-oxygenated androgens (69–71). ACTH also predicted A4 in males but not in females. These findings highlight potential sex-specific differences in steroidogenesis, HPA axis activity, gonadal involvement, and GC response. Broader prospective studies are therefore needed to clarify these mechanisms.

The findings of the current study also showed that hematological differences followed known sex patterns, with higher red blood cell counts and hemoglobin in males than in females, along with higher eosinophils and monocytes, whereas lymphocytes and neutrophils count showed no sex differences (72–74). Platelet counts, which are typically higher in females, did not differ significantly between males and females in the current cohort (75). Lipid parameters mirrored general population trends, with females showing higher total and HDL cholesterol levels and lower triglyceride levels (76), suggesting preserved estrogen-related cardio-protection. However, in less controlled cohorts, persistent androgen excess may offset this benefit, which should be an important consideration when evaluating the long-term impact of GC therapy.

This study has some limitations worth noting. First, the retrospective, cross-sectional design of this study limited causal inference. Second, hormonal measurements were conducted using immunoassays rather than liquid chromatography–tandem mass spectrometry, which could have introduced potential variability. However, analyses were performed in the laboratories of tertiary centers, and expressing hormone levels as ratios to the ULN helped mitigate inter-laboratory differences and allowed for standardized comparisons. Third, data on treatment adherence were not systematically collected, and subgroup analyses may be underpowered. Nonetheless, this study draws on a robust cohort of adult classic CAH patients from multiple Italian tertiary centers, enhancing the generalizability of results and reflecting real-life clinical practice. Importantly, this study provides a comprehensive analysis of sex-related differences in clinical, biochemical, metabolic, and hormonal features of classic CAH in adulthood.

## 5 Conclusions

This real-world analysis offers meaningful clinical insights into CAH management. In males, targeting metabolism, particularly visceral adiposity and glycemic control, may be crucial for improving hormonal control. If confirmed, these findings support prioritizing adiposity reduction and insulin-sensitizing strategies in CAH rather than relying solely on escalating GC doses to control androgen excess. The contribution of metabolic factors to disease dysregulation may be particularly relevant in males. In females, preserving the cardiometabolic protection of estrogen, especially in the context of good hormonal control, should remain a key focus of long-term care. Further prospective, longitudinal studies are certainly needed to confirm these findings and clarify their underlying pathophysiological mechanisms.

## Data Availability

The raw data supporting the conclusions of this article will be made available by the authors, without undue reservation.

## References

[1] Auer MK Nordenstrom A Lajic S Reisch N . Congenital adrenal hyperplasia. Lancet. (2023) 401:227–44. doi: 10.1530/ey.20.6.14 36502822

[2] Merke DP Auchus RJ . Congenital adrenal hyperplasia due to 21-hydroxylase deficiency. N Engl J Med. (2020) 383:1248–61. doi: 10.1530/ey.18.8.16 32966723

[3] Grumbach MM Shaw EB . Further studies on the treatment of congenital adrenal hyperplasia with cortisone: IV. Effect of cortisone and compound B in infants with disturbed electrolyte metabolism, by John F. Crigler Jr, MD, Samuel H. Silverman, MD, and Lawson Wilkins, MD, Pediatrics, 1952;10:397-413. Pediatrics. (1998) 102:215–21. doi: 10.1542/peds.102.s1.215 9651433

[4] Pofi R Ji X Krone NP Tomlinson JW . Long-term health consequences of congenital adrenal hyperplasia. Clin Endocrinol (Oxf). (2024) 101:318–31. doi: 10.1111/cen.14967 37680029

[5] Bowden SA Henry R . Pediatric adrenal insufficiency: Diagnosis, management, and new therapies. Int J Pediatr. (2018) 2018:1739831. doi: 10.1155/2018/1739831 30515225 PMC6236909

[6] Perry R Kecha O Paquette J Huot C Van Vliet G Deal C . Primary adrenal insufficiency in children: Twenty years experience at the Sainte-Justine Hospital, Montreal. J Clin Endocrinol Metab. (2005) 90:3243–50. doi: 10.1210/jc.2004-0016 15811934

[7] Mehmood KT Rentea RM . Ambiguous genitalia and disorders of sexual differentiation. In: StatPearls. Treasure Island (FL (2025). 32491367

[8] Banerjee S Bajpai A . Precocious puberty. Indian J Pediatr. (2023) 90:582–9. doi: 10.1007/s12098-023-04554-4 37074536

[9] Bretones P Riche B Pichot E David M Roy P Tardy V . Growth curves for congenital adrenal hyperplasia from a national retrospective cohort. J Pediatr Endocrinol Metab. (2016) 29:1379–88. doi: 10.1515/jpem-2016-0156 27852974

[10] Muthusamy K Elamin MB Smushkin G Murad MH Lampropulos JF Elamin KB . Clinical review: Adult height in patients with congenital adrenal hyperplasia: A systematic review and metaanalysis. J Clin Endocrinol Metab. (2010) 95:4161–72. doi: 10.1210/jc.2009-2616 20823467

[11] Pereira N Lin-Su K . Reproductive function and fertility in women with congenital adrenal hyperplasia. EMJ Repro Health. (2018) 2018:101–7. doi: 10.33590/emjreprohealth/10314092

[12] Lobo RA Goebelsmann U . Adult manifestation of congenital adrenal hyperplasia due to incomplete 21-hydroxylase deficiency mimicking polycystic ovarian disease. Am J Obstet Gynecol. (1980) 138:720–6. doi: 10.1016/0002-9378(80)90095-2 6254362

[13] Pall M Azziz R Beires J Pignatelli D . The phenotype of hirsute women: A comparison of polycystic ovary syndrome and 21-hydroxylase-deficient nonclassic adrenal hyperplasia. Fertil Steril. (2010) 94:684–9. doi: 10.1016/j.fertnstert.2009.06.025 19726039

[14] Engels M Span PN van Herwaarden AE Sweep F Stikkelbroeck N Claahsen-van der Grinten HL . Testicular adrenal rest tumors: Current insights on prevalence, characteristics, origin, and treatment. Endocr Rev. (2019) 40:973–87. doi: 10.1210/er.2018-00258 30882882

[15] Falhammar H Nystrom HF Ekstrom U Granberg S Wedell A Thoren M . Fertility, sexuality and testicular adrenal rest tumors in adult males with congenital adrenal hyperplasia. Eur J Endocrinol. (2012) 166:441–9. doi: 10.1530/eje-11-0828 22157069 PMC3290120

[16] Stikkelbroeck NM Otten BJ Pasic A Jager GJ Sweep CG Noordam K . High prevalence of testicular adrenal rest tumors, impaired spermatogenesis, and Leydig cell failure in adolescent and adult males with congenital adrenal hyperplasia. J Clin Endocrinol Metab. (2001) 86:5721–8. doi: 10.1210/jcem.86.12.8090 11739428

[17] Tresoldi AS Betella N Hasenmajer V Pozza C Vena W Fiamengo B . Bilateral testicular masses and adrenal insufficiency: Is congenital adrenal hyperplasia the only possible diagnosis? First two cases of TARTS described in Addison-only X-linked adrenoleukodystrophy and a brief review of literature. J Endocrinol Invest. (2021) 44:391–402. doi: 10.1007/s40618-020-01362-x 32691371

[18] Bouvattier C Esterle L Renoult-Pierre P de la Perriere AB Illouz F Kerlan V . Clinical outcome, hormonal status, gonadotrope axis, and testicular function in 219 adult men born with classic 21-hydroxylase deficiency. A French national survey. J Clin Endocrinol Metab. (2015) 100:2303–13. doi: 10.1210/jc.2014-4124 25822101

[19] De Alcubierre D Ferrari D Mauro G Isidori AM Tomlinson JW Pofi R . Glucocorticoids and cognitive function: A walkthrough in endogenous and exogenous alterations. J Endocrinol Invest. (2023) 46:1961–82. doi: 10.1007/s40618-023-02091-7 37058223 PMC10514174

[20] Barbot M Mazzeo P Lazzara M Ceccato F Scaroni C . Metabolic syndrome and cardiovascular morbidity in patients with congenital adrenal hyperplasia. Front Endocrinol (Lausanne). (2022) 13:934675. doi: 10.3389/fendo.2022.934675 35979433 PMC9376294

[21] Krysiak R Claahsen-van der Grinten HL Reisch N Touraine P Falhammar H . Cardiometabolic aspects of congenital adrenal hyperplasia. Endocr Rev. (2025) 46:80–148. doi: 10.1210/endrev/bnae026 39240753 PMC11720181

[22] Ekbom K Strandqvist A Lajic S Hirschberg A Falhammar H Nordenstrom A . The impact of adherence and therapy regimens on quality of life in patients with congenital adrenal hyperplasia. Clin Endocrinol (Oxf). (2022) 96:666–79. doi: 10.1111/cen.14676 34994970 PMC9303581

[23] Ridder LO Balle CM Skakkebaek A Lind-Holst M Nielsen MM Hermann P . Endocrine, cardiac and neuropsychological aspects of adult congenital adrenal hyperplasia. Clin Endocrinol (Oxf). (2024) 100:515–26. doi: 10.1111/cen.15055 38572909

[24] Paizoni L Auer MK Schmidt H Hubner A Bidlingmaier M Reisch N . Effect of androgen excess and glucocorticoid exposure on metabolic risk profiles in patients with congenital adrenal hyperplasia due to 21-hydroxylase deficiency. J Steroid Biochem Mol Biol. (2020) 197:105540. doi: 10.1016/j.jsbmb.2019.105540 31730799

[25] Tamhane S Rodriguez-Gutierrez R Iqbal AM Prokop LJ Bancos I Speiser PW . Cardiovascular and metabolic outcomes in congenital adrenal hyperplasia: A systematic review and meta-analysis. J Clin Endocrinol Metab. (2018) 103:4097–103. doi: 10.1210/jc.2018-01862 30272185

[26] Speiser PW Arlt W Auchus RJ Baskin LS Conway GS Merke DP . Congenital adrenal hyperplasia due to steroid 21-hydroxylase deficiency: An endocrine society clinical practice guideline. J Clin Endocrinol Metab. (2018) 103:4043–88. doi: 10.1530/ey.16.8.5 30272171 PMC6456929

[27] World Health Organization . Obesity: Preventing and managing the global epidemic. Report of a WHO consultation. World Health Organ Tech Rep Ser. (2000) 894:i–xii, 1–253. 11234459

[28] Motamed N Perumal D Zamani F Ashrafi H Haghjoo M Saeedian FS . Conicity index and waist-to-hip ratio are superior obesity indices in predicting 10-year cardiovascular risk among men and women. Clin Cardiol. (2015) 38:527–34. doi: 10.1002/clc.22437 26418518 PMC6490781

[29] Valdez R . A simple model-based index of abdominal adiposity. J Clin Epidemiol. (1991) 44:955–6. doi: 10.1016/0895-4356(91)90059-i 1890438

[30] Valdez R Seidell JC Ahn YI Weiss KM . A new index of abdominal adiposity as an indicator of risk for cardiovascular disease. A cross-population study. Int J Obes Relat Metab Disord. (1993) 17:77–82. doi: 10.1163/ej.9789004191921.i-344.45 8384168

[31] Kahn HS . The "lipid accumulation product" performs better than the body mass index for recognizing cardiovascular risk: A population-based comparison. BMC Cardiovasc Disord. (2005) 5:26. doi: 10.1186/1471-2261-5-26 16150143 PMC1236917

[32] Cho S Shin A Choi JY Park SM Kang D Lee JK . Optimal cutoff values for anthropometric indices of obesity as discriminators of metabolic abnormalities in Korea: Results from a Health Examinees study. BMC Public Health. (2021) 21:459. doi: 10.1186/s12889-021-10490-9 33676466 PMC7937287

[33] Matthews DR Hosker JP Rudenski AS Naylor BA Treacher DF Turner RC . Homeostasis model assessment: Insulin resistance and beta-cell function from fasting plasma glucose and insulin concentrations in man. Diabetologia. (1985) 28:412–9. doi: 10.1007/bf00280883 3899825

[34] WHO . Infertility. Available online at: https://www.who.int/news-room/fact-sheets/detail/infertility (Accessed March 6, 2026).

[35] Practice Committee of the American Society for Reproductive Medicine ASfRMWDC . Definition of infertility: A committee opinion. Fertil Steril. (2023) 120:1170. doi: 10.1016/s0029-7844(02)01986-5 40991339

[36] Isidori AM Aversa A Calogero A Ferlin A Francavilla S Lanfranco F . Adult- and late-onset male hypogonadism: The clinical practice guidelines of the Italian Society of Andrology and Sexual Medicine (SIAMS) and the Italian Society of Endocrinology (SIE). J Endocrinol Invest. (2022) 45:2385–403. doi: 10.1007/s40618-022-01859-7 36018454 PMC9415259

[37] Corona G Goulis DG Huhtaniemi I Zitzmann M Toppari J Forti G . European Academy of Andrology (EAA) guidelines on investigation, treatment and monitoring of functional hypogonadism in males: Endorsing organization: European Society of Endocrinology. Andrology. (2020) 8:970–87. doi: 10.1111/andr.12770 32026626

[38] Auchus RJ . Management considerations for the adult with congenital adrenal hyperplasia. Mol Cell Endocrinol. (2015) 408:190–7. doi: 10.1016/j.mce.2015.01.039 25643980

[39] Auchus RJ Sarafoglou K Fechner PY Vogiatzi MG Imel EA Davis SM . Crinecerfont lowers elevated hormone markers in adults with 21-hydroxylase deficiency congenital adrenal hyperplasia. J Clin Endocrinol Metab. (2022) 107:801–12. doi: 10.1530/ey.19.8.8 34653252 PMC8851935

[40] Khattab A Charlton RW . Corticotropin releasing factor-1 receptor antagonism associated with favorable outcomes of male reproductive health biochemical parameters. Front Endocrinol (Lausanne). (2023) 14:1127558. doi: 10.3389/fendo.2023.1127558 37284216 PMC10241302

[41] Roszkowska Z Bobrowicz M Betlejewska J Hubska J Rak-Makowska B Ambroziak U . Fertility in congenital adrenal hyperplasia due to 21-hydroxylase deficiency: A review. Front Endocrinol (Lausanne). (2025) 16:1682341. doi: 10.3389/fendo.2025.1682341 41393299 PMC12698369

[42] Sarafoglou K Merke DP Reisch N Claahsen-van der Grinten H Falhammar H Auchus RJ . Interpretation of steroid biomarkers in 21-hydroxylase deficiency and their use in disease management. J Clin Endocrinol Metab. (2023) 108:2154–75. doi: 10.1530/ey.20.12.8 36950738 PMC10438890

[43] Auchus RJ Hamidi O Pivonello R Bancos I Russo G Witchel SF . Phase 3 trial of crinecerfont in adult congenital adrenal hyperplasia. N Engl J Med. (2024) 391:504–14. doi: 10.1056/nejmoa2404656 38828955 PMC11309900

[44] Kim SH Han S Zhao J Wang S Kusnetzow AK Reinhart G . Discovery of CRN04894: A novel potent selective MC2R antagonist. ACS Med Chem Lett. (2024) 15:478–85. doi: 10.1021/acsmedchemlett.3c00514 38628803 PMC11017392

[45] Lu CY Terry V Thomas DM . Precision medicine: Affording the successes of science. NPJ Precis Oncol. (2023) 7:3. doi: 10.1038/s41698-022-00343-y 36599878 PMC9812011

[46] Marcozzi B Lo Noce C Vannucchi S Di Lonardo A Damiano C Galeone D . Measured obesity and overweight in adults: The Italian Health Examination Survey 2023-CUORE Project. Eur J Public Health. (2024) 34:iii560–1. doi: 10.1093/eurpub/ckae144.1441 39659294

[47] Collaborators GBDAB . Global, regional, and national prevalence of adult overweight and obesity, 1990-2021, with forecasts to 2050: A forecasting study for the Global Burden of Disease Study 2021. Lancet. (2025) 405:813–38. doi: 10.1016/S0140-6736(25)00355-1, PMID: 40049186 PMC11920007

[48] Borges JH de Oliveira DM de Lemos-Marini SHV Geloneze B Goncalves EM Guerra-Junior G . Fat distribution and lipid profile of young adults with congenital adrenal hyperplasia due to 21-hydroxylase enzyme deficiency. Lipids. (2021) 56:101–10. doi: 10.1002/lipd.12280 32929736

[49] Halper A Sanchez B Hodges JS Kelly AS Dengel D Nathan BM . Bone mineral density and body composition in children with congenital adrenal hyperplasia. Clin Endocrinol (Oxf). (2018) 88:813–9. doi: 10.1111/cen.13580 29460378 PMC5980722

[50] Charoensri S Auchus RJ . Predictors of cardiovascular morbidities in adults with 21-hydroxylase deficiency congenital adrenal hyperplasia. J Clin Endocrinol Metab. (2024) 109:e1133–42. doi: 10.1210/clinem/dgad628 37878953

[51] El-Maouche D Hannah-Shmouni F Mallappa A Hargreaves CJ Avila NA Merke DP . Adrenal morphology and associated comorbidities in congenital adrenal hyperplasia. Clin Endocrinol (Oxf). (2019) 91:247–55. doi: 10.1111/cen.13996 31001843 PMC6635023

[52] Busetto L Dicker D Fruhbeck G Halford JCG Sbraccia P Yumuk V . A new framework for the diagnosis, staging and management of obesity in adults. Nat Med. (2024) 30:2395–9. doi: 10.1038/s41591-024-03095-3 38969880

[53] Ashwell M Gunn P Gibson S . Waist-to-height ratio is a better screening tool than waist circumference and BMI for adult cardiometabolic risk factors: systematic review and meta-analysis. Obes Rev. (2012) 13:275–86. doi: 10.1111/j.1467-789x.2011.00952.x 22106927

[54] Werdermann M Berger I Scriba LD Santambrogio A Schlinkert P Brendel H . Insulin and obesity transform hypothalamic-pituitary-adrenal axis stemness and function in a hyperactive state. Mol Metab. (2021) 43:101112. doi: 10.1016/j.molmet.2020.101112 33157254 PMC7691554

[55] Incollingo Rodriguez AC Epel ES White ML Standen EC Seckl JR Tomiyama AJ . Hypothalamic-pituitary-adrenal axis dysregulation and cortisol activity in obesity: a systematic review. Psychoneuroendocrinology. (2015) 62:301–18. doi: 10.1016/j.psyneuen.2015.08.014 26356039

[56] Lawrence NR Dawson J Lang ZQ Prete A Baranowski ES Schiffer L . Modelling adrenal steroid profiles to inform monitoring guidance in congenital adrenal hyperplasia. EBioMedicine. (2025) 116:105749. doi: 10.1016/j.ebiom.2025.105749 40398353 PMC12148605

[57] Arner P . Regional adipocity in man. J Endocrinol. (1997) 155:191–2. doi: 10.1677/joe.0.1550191 9415044

[58] Karastergiou K Smith SR Greenberg AS Fried SK . Sex differences in human adipose tissues - the biology of pear shape. Biol Sex Differ. (2012) 3:13. doi: 10.1186/2042-6410-3-13 22651247 PMC3411490

[59] Mauvais-Jarvis F Clegg DJ Hevener AL . The role of estrogens in control of energy balance and glucose homeostasis. Endocr Rev. (2013) 34:309–38. doi: 10.1210/er.2012-1055 23460719 PMC3660717

[60] Pasquali R Oriolo C . Obesity and androgens in women. Front Horm Res. (2019) 53:120–34. doi: 10.1159/000494908 31499497

[61] Nikolic M Macut D Djordjevic A Velickovic N Nestorovic N Bursac B . Possible involvement of glucocorticoids in 5alpha-dihydrotestosterone-induced PCOS-like metabolic disturbances in the rat visceral adipose tissue. Mol Cell Endocrinol. (2015) 399:22–31. doi: 10.1016/j.mce.2014.08.013, PMID: 25179821

[62] Dakin RS Walker BR Seckl JR Hadoke PW Drake AJ . Estrogens protect male mice from obesity complications and influence glucocorticoid metabolism. Int J Obes (Lond). (2015) 39:1539–47. doi: 10.1038/ijo.2015.102 26032810 PMC4564952

[63] Andersson T Soderstrom I Simonyte K Olsson T . Estrogen reduces 11beta-hydroxysteroid dehydrogenase type 1 in liver and visceral, but not subcutaneous, adipose tissue in rats. Obes (Silver Spring). (2010) 18:470–5. doi: 10.1038/oby.2009.294 19763091

[64] Yamatani H Takahashi K Yoshida T Takata K Kurachi H . Association of estrogen with glucocorticoid levels in visceral fat in postmenopausal women. Menopause. (2013) 20:437–42. doi: 10.1097/gme.0b013e318271a640 23149864

[65] Li S Tao T Wang L Mao X Zheng J Zhao A . The expression of 11beta-HSDs, GR, and H6PDH in subcutaneous adipose tissue from polycystic ovary syndrome subjects. Horm Metab Res. (2013) 45:802–7. doi: 10.1055/s-0033-1345186 23979790

[66] Kroon J Pereira AM Meijer OC . Glucocorticoid sexual dimorphism in metabolism: dissecting the role of sex hormones. Trends Endocrinol Metab. (2020) 31:357–67. doi: 10.1016/j.tem.2020.01.010 32037025

[67] Mapas-Dimaya AC Agdere L Bahtiyar G Mejia JO Sacerdote AS . Metformin-responsive classic salt-losing congenital adrenal hyperplasia due to 21-hydroxylase deficiency: a case report. Endocr Pract. (2008) 14:889–91. doi: 10.4158/ep.14.7.889 18996819

[68] Rezvani I Garibaldi LR Digeorge AM Artman HG . Disproportionate suppression of dehydroepiandrosterone sulfate (DHEAS) in treated patients with congenital adrenal hyperplasia due to 21-hydroxylase deficiency. Pediatr Res. (1983) 17:131–4. doi: 10.1203/00006450-198302000-00010 6219334

[69] Sarafoglou K Auchus RJ . Future directions in the management of classic congenital adrenal hyperplasia due to 21-hydroxylase deficiency. J Clin Endocrinol Metab. (2025) 110:S74–87. doi: 10.1210/clinem/dgae759 39836617 PMC11749912

[70] Turcu AF Rege J Auchus RJ Rainey WE . 11-Oxygenated androgens in health and disease. Nat Rev Endocrinol. (2020) 16:284–96. doi: 10.1530/ey.17.8.20 32203405 PMC7881526

[71] Mallappa A Merke DP . Management challenges and therapeutic advances in congenital adrenal hyperplasia. Nat Rev Endocrinol. (2022) 18:337–52. doi: 10.1530/ey.19.8.16 35411073 PMC8999997

[72] Wakeman L Al-Ismail S Benton A Beddall A Gibbs A Hartnell S . Robust, routine haematology reference ranges for healthy adults. Int J Lab Hematol. (2007) 29:279–83. doi: 10.1111/j.1365-2257.2006.00883.x 17617078

[73] Bain BJ . Ethnic and sex differences in the total and differential white cell count and platelet count. J Clin Pathol. (1996) 49:664–6. doi: 10.1136/jcp.49.8.664 8881919 PMC500612

[74] Buoro S . Determinazione degli intervalli di riferimento dell’esame emocromocitometrico eseguito con analizzatore Sysmex XN 9000. Biochim Clinica. (2015) 39:259.

[75] Biino G Santimone I Minelli C Sorice R Frongia B Traglia M . Age- and sex-related variations in platelet count in Italy: a proposal of reference ranges based on 40987 subjects' data. PloS One. (2013) 8:e54289. doi: 10.1371/journal.pone.0054289 23382888 PMC3561305

[76] Holven KB Roeters van Lennep J . Sex differences in lipids: a life course approach. Atherosclerosis. (2023) 384:117270. doi: 10.1016/j.atherosclerosis.2023.117270 37730457

