# Supplementary material for: Sex differences in classic congenital adrenal hyperplasia: a multicenter, real-world analysis
Source: Front Endocrinol (Lausanne). 2026 Apr 27;17:1788502. doi: 10.3389/fendo.2026.1788502 (PMC13158096; doi:10.3389/fendo.2026.1788502)
Supplement: Supplementary file 1 [file DataSheet1.docx]

**Supplementary File 1: Details on used immunoassays for hormone measurement.**

**“Federico II” University of Naples**

Plasma renin was measured using a chemiluminescent immunoassay on the IDS‑iSYS Multi‑Discipline Automated Analyzer (Immunodiagnostic Systems, Boldon, UK).

Plasma ACTH and serum A4 were measured using chemiluminescent immunometric assays on the IMMULITE 2000 Immunoassay System (Siemens Healthineers, Erlangen, Germany).

Serum 17OHP was measured using a chemiluminescent immunoassay on the IDS‑iSYS Multi‑Discipline Automated Analyzer.

Serum total testosterone and DHEAS were measured by chemiluminescent immunoassays on the Atellica IM Analyzer (Siemens Healthineers, Erlangen, Germany).

**“Sapienza" University of Rome**

Plasma ACTH was measured by immunoradiometric assay (IRMA) using Beckman Coulter reagents (ref. IM2030, B89463). EDTA plasma samples were collected and processed as soon as possible by centrifugation at 2–8 °C. Due to the instability of ACTH, plasma samples were stored at −18 °C when the assay was not performed immediately.

Plasma renin, serum 17OHP, and A4 were measured by radioimmunoassay (RIA) using Beckman Coulter reagents (Beckman Coulter, Brea, CA, USA).

Serum DHEAS was measured using a chemiluminescent microparticle immunoassay (CMIA) on the Abbott ARCHITECT i System with ARCHITECT DHEAS reagent (ref. 8K27).

**“Sant'Andrea” University Hospital of Rome**

Plasma ACTH was measured using a chemiluminescent immunoassay with the LIAISON ACTH assay (DiaSorin, Saluggia, Italy).

Plasma renin activity was measured by enzyme-linked immunosorbent assay (ELISA) using kits from Labor Diagnostika Nord (Nordhorn, Germany).

Serum steroid profiling (including total testosterone, A4, DHEAS and 17OHP) was performed by liquid chromatography–tandem mass spectrometry (LC-MS/MS) using the MassChrom Steroids in Serum/Plasma kit (Chromsystems Instruments & Chemicals GmbH, Gräfelfing, Germany), allowing the simultaneous quantification of 15 steroids

**“G. Martino" University Hospital of Messina**

Plasma ACTH, serum total testosterone, and dehydroepiandrosterone sulfate (DHEAS) were measured using electrochemiluminescence immunoassays (ECLIA) on the Cobas immunoassay analyzers (Roche Diagnostics, Mannheim, Germany).

Serum Δ4-androstenedione (A4), 17-hydroxyprogesterone (17OHP), and renin were measured by enzyme-linked immunosorbent assays (ELISA) using kits from DiaSource ImmunoAssays (Louvain-la-Neuve, Belgium).
