# Supplementary material for: Sex differences in classic congenital adrenal hyperplasia: a multicenter, real-world analysis
Source: Front Endocrinol (Lausanne). 2026 Apr 27;17:1788502. doi: 10.3389/fendo.2026.1788502 (PMC13158096; doi:10.3389/fendo.2026.1788502)
Supplement: Supplementary file 2 [file Table1.docx]

| **Supplementary Table 1.** Sex comparison of metabolic and hormonal phenotypes across salt-wasting (SW) and simple-virilazing (SV) CAH. | | | | | | | |
| --- | --- | --- | --- | --- | --- | --- | --- |
|  | **SW-CAH (N=87)** | | |  | **SV-CAH (N=36)** | | |
|  | **Male patients**  (n=38) | **Female patients**  (n= 49) | **p-value** |  | **Male patients**  (n=8) | **Female patients**  (n=28) | **p-value** |
| Age (years) | 31.0 (22.0-40.3) | 32.0 (23.0-37.0) | 0.891 |  | 36.5 (28.3-56.0) | 30.0 (24.3-36.8) | 0.135 |
| Disease duration (years) | 31.0 (21.8-39.3) | 31.0 (22.0-37.0) | 0.949 |  | 29.0 (20.0-35.0) | 23.0 (15.3-30.4) | 0.199 |
| BSA-adjusted HCeq daily GC dose (mg/m^2^) | 15.2 (11.6-18.9) | 14.0 (11.4-18.3) | 0.817 |  | 13.0 (9.7-14.4) | 12.4 (6.7-17.2) | 0.765 |
| **Anthropometric measures** |  |  |  |  |  |  |  |
| BMI (Kg/m^2^) | 27.0 (24.5-31.8) | 24.2 (22.5-27.7) | **0.003** |  | 26.1 (24.8-29.2) | 25.0 (21.7-30.4) | 0.550 |
| Waist Circumference (cm) | 94 (86-102) | 78 (74-90) | **<0.001** |  | 95 (80-99) | 76 (72-91) | 0.063 |
| WHtR | 0.55 (0.50-0.61) | 0.51 (0.46-0.55) | **0.011** |  | 0.54 (0.47-0.59) | 0.48 (0.44-0.53) | 0.310 |
| LAP | 24.1 (13.3-37.9) | 14.6 (10.6-25.0) | **0.015** |  | 18.5 (9.6-53.4) | 14.7 (7.8-28.7) | 0.733 |
| C-index | 1.25 (1.16-1.32) | 1.18 (1.12-1.24) | **0.015** |  | 1.28 (1.13-1.32) | 1.15 (1.10-1.24) | **0.035** |
| **Comorbidities** | | | | | | | |
| Obesity (BMI ≥ 30 Kg/m2) | 35.1% | 14.6% | **0.025** |  | 25.0% | 29.6% | 0.589 |
| Overweight (25 Kg/m2 ≤ BMI < 30 Kg/m2) | 40.5% | 29.2% | 0.193 |  | 50.0% | 22.2% | 0.140 |
| Overweight or Obesity (BMI ≥25 Kg/m2) | 75.7% | 43.8% | **0.003** |  | 75.0% | 51.9% | 0.228 |
| **Glucose and lipid metabolism*** |  |  |  |  |  |  |  |
| Fasting plasma Glucose (mg/dL) | 85.3 ± 9.9 | 83.3 ± 9.6 | 0.376 |  | 86.5 ± 7.9 | 81.2 ± 9.1 | 0.150 |
| Fasting Insulin (mUI/mL) | 10.3 (6.2-14.1) | 8.8 (5.6-13.3) | 0.388 |  | 9.9 (5.4-10.3) | 10.3 (7.0-13.9) | 0.217 |
| HOMA-IR | 2.18 (1.17-2.70) | 1.74 (0.95-2.66) | 0.276 |  | 1.96 (0.92-2.20) | 2.01 (1.37-3.08) | 0.335 |
| HbA1c, (mmol/mol) | 33 (30-37) | 32 (31-33) | 0.065 |  | 33 (32-36) | 33 (32-37) | 0.894 |
| Total Cholesterol, (mg/dL) | 166 ± 31 | 177 ± 31 | 0.122 |  | 168 ± 31 | 182 ± 29 | 0.312 |
| LDL Cholesterol, (mg/dL) | 100 (80-116) | 89 (75-115) | 0.276 |  | 99 (82-113) | 97 (85-111) | 0.739 |
| HDL Cholesterol, (mg/dL) | 48 (39-63) | 56 (49-78) | **0.001** |  | 47 (36-69) | 67 (57-78) | **0.031** |
| Triglycerides (mg/dL) | 87 (68-105) | 66 (52-88) | **0.039** |  | 87 (62-153) | 80 (58-102) | 0.505 |
| **Hormones** |  |  |  |  |  |  |  |
| 17OH-Progesterone (ng/mL) | 14.35 (4.55-25.31) | 5.19 (1.3-14.14) | **0.003** |  | 17.7 (4.8-21.0) | 3.5 (1.5-14.5) | 0.093 |
| Androstenedione / ULN | 0.55 (0.16-1.19) | 0.30 (0.13-0.68) | **0.044** |  | 0.57 (0.26-1.18) | 0.60 (0.22-1.13) | 0.603 |
| DHEAS / ULN | 0.14 (0.07-0.20) | 0.04 (0.02-0.16) | **0.009** |  | 0.17 (0.06-0.48) | 0.17 (0.07-0.39) | 0.813 |
| ACTH / ULN | 1.97 (0.47-4.13) | 1.12 (0.26-7.63) | 0.991 |  | 0.77 (0.57-1.40) | 0.29 (0.11-0.94) | 0.070 |
| Testosterone / ULN | 0.50 (0.34-0.61) | 0.36 (0.14-0.84) | 0.221 |  | 0.42 (0.30-0.71) | 0.48 (0.16-0.78) | 0.984 |

Data are presented as mean±SD or median (IQR25-75) according to distribution. *Excluding patients with diabetes mellitus.

BSA, body surface area; HCeq, hydrocortisone equivalnce; GC, glucocorticoid; BMI, body mass index; WHtR, waist-to-height ratio; LAP, lipid accumulation product; C-index, conicity index; HOMA-IR, homestatic model assesment-insulin resistance; HbA1c, glycated hemoglobin; LDL, low density lipoprotein; HDL, high density lipoprotein; DHEAS, dehydroepiandrosterone sulfate; ACTH, adrenocorticotropic hormone; ULN, upper limit of normal.
